# Pacemaker Implantation for Low‐Grade Conduction Abnormalities After Balloon‐Expandable Transcatheter Aortic Valve Implantation

**DOI:** 10.1002/clc.70028

**Published:** 2024-10-24

**Authors:** Julian Wolfes, Fernando de Torres Alba, Gerrit Kaleschke, Julia Vormbrock, Florian Reinke, Christian Ellermann, Helmut Baumgartner, Lars Eckardt, Gerrit Frommeyer

**Affiliations:** ^1^ Department of Cardiology II—Electrophysiology University Hospital Münster Münster Germany; ^2^ Department of Cardiology III—Adult Congenital and Valvular Heart Disease University Hospital Münster Münster Germany

**Keywords:** low grade conduction disorders, pacemaker indication, TAVR

## Abstract

**Introduction:**

A frequent complication after TAVI are postinterventional conduction abnormalities requiring permanent pacemaker implantation. In this study, we analyzed the characteristics of borderline conduction abnormalities leading to pacemaker implantation and the resulting ventricular pacing amounts.

**Methods and Results:**

All patients who underwent balloon‐expandable TAVI between 2014 and 2019 in our tertiary center were analyzed in a retrospective manner. One hundred and sixty‐five patients of 1083 TAVI‐patients developed postinterventional conduction abnormalities leading to pacemaker implantation. Of these 19 (11.5%) did not represent a clear indication for cardiac pacing according to current European guidelines. Patient characteristics, underlying conduction abnormalities, and the temporal change of ventricular pacing percentages at 24 h and 6 weeks after pacemaker implantation were analyzed.

The dominating borderline conduction abnormalities leading to pacemaker implantation were new‐onset persisting bundle‐branch‐blocks and new first‐degree AV‐blocks with progression of AV‐delay.

While pacemaker implantation was safe and without severe complications in all cases, only 6 of 19 patients had high pacing amounts (95%−100%) after 24 h while 11 patients had low to no pacing amounts (0%−5%). After 6 weeks, 8 patients showed decreasing pacing amounts, no patient had an increasing amount of ventricular pacing and all patients had an intrinsic ventricular rhythm > 30/min.

**Conclusion:**

In our cohort of 1038 TAVI patients, 19 patients underwent PMI for borderline CAs (11.5% of all PMI). Of these, only 2 patients had high pacing amounts after 6 weeks. The risk of complete persisting heart block in these patients is very low. Furthermore, algorithms to reduce ventricular pacing are highly effective to avoid ventricular pacing whenever reasonable.

## Graphical abstract

Graphical abstract summarizing the key features of the study.

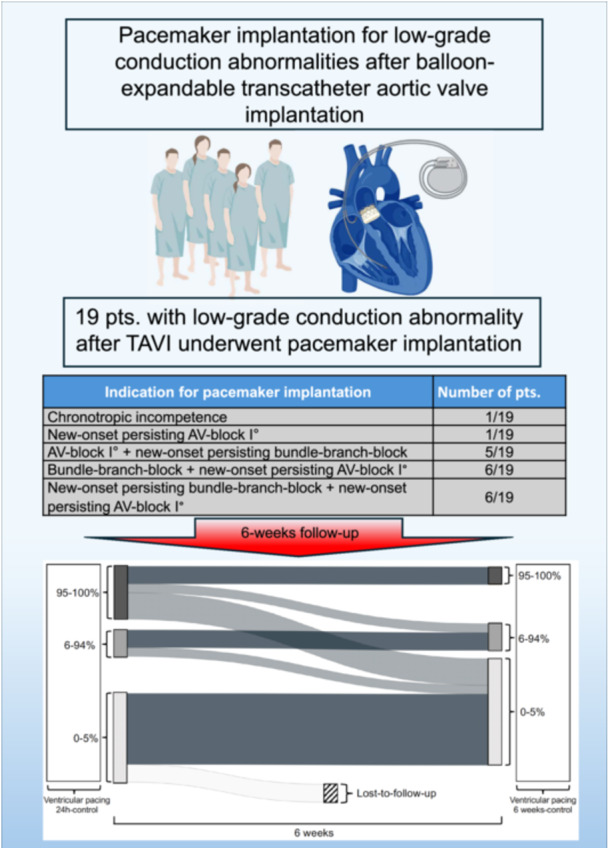

## 1 Introduction

Transcatheter aortic valve implantation (TAVI) is an established therapy for severe aortic stenosis in patients at high or intermediate operative risk [1] and extensions of indications are under investigation [1, 2, 3, 4]. While previous studies have shown that TAVI reduces mortality, cardiac symptoms, and rehospitalization [5], a major concern of TAVI remains peri‐ and postprocedural conduction abnormalities (CA) directly after or in the first days following the procedure. Many studies focused on preprocedural predictors of severe CAs [6, 7] and the time course of their onset [8].

When focusing on the type of CAs occurring after TAVI, significant CA with clear consecutive pacemaker indications are dominating and represent about three‐quarters of the reasons leading to pacemaker implantation (PMI) [8, 9]. Nevertheless, about a quarter of TAVI patients undergo PMI for CAs which do not represent Class I or IIa PM indications according to the European Society of Cardiology (ESC) Guidelines on cardiac pacing [10]. These postinterventional CAs, which may also be significant and, for example, represent progressive AV‐delays and/or new‐onset bundle‐branch‐blocks (BBB), are often concerning and the safety of a discharge strategy without PMI in these patients is questioned.

Previous studies that analyzed these patients came to heterogeneous conclusions concerning pacemaker dependence. The study by Mirolo et al. [9] seems to favor PMI based on the analysis of pacemaker memories. On the other hand, Ramazzina et al. [11] do not see a need for pacing derived from the PM memories of these borderline‐indication patients. Notably, the above‐mentioned studies used different modes of PM programming with short AV‐intervals and no algorithms to reduce ventricular pacing in the study by Mirolo et al. To help clarify decision‐making on PM implantation in this specific patient group, we investigated PM implantations in borderline indication patients after TAVI with a widely used balloon‐expandable valve.

## 2 Methods

### 2.1 Patient Selection

All TAVI patients signed an informed consent for interventional treatment and data collection allowing intra‐institutional retrospective data analysis. The study was approved by the local ethics committee and complies with the Declaration of Helsinki. All patients who underwent TAVI with the balloon‐expandable Edwards Sapien 3 valve (Edwards Lifesciences, Irvine, CA, USA) at our tertiary center between January 2014 and December 2019 were included in the preselection. Patients who died during the procedure and those with a PM or a transvenous cardioverter‐defibrillator implanted before TAVI were excluded for further analysis. Thorough clinical and echocardiographic evaluation was mandatory before the indication for TAVI was established by the multidisciplinary heart team, including noninvasive and invasive cardiologists and cardiac surgeons. All TAVI patients signed an informed consent for interventional treatment and data collection allowing intra‐institutional retrospective data analysis. The study was approved by the local ethics committee and complies with the Declaration of Helsinki.

### 2.2 ECG Analysis and PM Indication

All available clinical records before during and after TAVI were prospectively collected and analyzed. Patients were monitored for at least 7 days after TAVI with continuous 3‐lead ECG telemetry (Infinity M300, Dräger, Lübeck, Germany); furthermore, daily 12‐lead ECGs were manually analyzed.

Indication for pacemakers after TAVI was established by an interdisciplinary team including electrophysiologists, cardiac surgeons, and the valvular interventional team. In this study, only patients with borderline indications according to the ESC guidelines [12] were included.

Pacing mode was chosen by the electrophysiologist responsible for the PMI and algorithms to reduce ventricular pacing were chosen whenever considered reasonable. All pacemakers were read after 24 h and 6 weeks following implantation and pacing amounts were integrated into the database. Furthermore, all patients were scheduled for a 1‐year follow‐up including echocardiography to evaluate valve function and the temporal change of left ventricular ejection fraction among other parameters.

## 3 Results

During the study period, 1254 patients underwent TAVI with an Edwards S3 valve. Seven patients died during the procedure and 164 patients were excluded from analysis because of a previous pacemaker or transvenous ICD implantation. One hundred and sixty‐five patients developed an indication for PMI after TAVI of which 146 patients had a Class I or IIa indication according to the ESC guidelines [10]. Nineteen patients underwent PMI for borderline indications based on expert consensus decision beyond guideline positions.

### 3.1 CA Before TAVI

The patient characteristics are shown in Table 1. Of 19 patients who underwent PMI for borderline indications, 12 patients had previous CA. Five patients had previous first‐degree AV‐block (AV‐I) ranging between 210 and 240 ms, 2 patients had a right bundle‐branch‐block (RBBB), 1 patient had combined AV‐I and RBBB, 3 patients had a previous LBBB, and 1 patient had a previous left anterior hemiblock (LAH).

**Table 1:** Patient characteristics at baseline.

Age (years)	82.6 ± 4.4
Female (%)	11 patients (63.2%)
EuroScore	17.7 ± 15.1
Ejection fraction (%)	55.7 ± 9.4
Preexisting CAs	12 patients (63%)
First‐degree AV‐block	5 patients (26%)
Right bundle branch block	2 patients (11%)
RBBB and first‐degree AV‐block	1 patient (5%)
Left bundle branch block	3 patients (16%)
Left anterior hemiblock	1 patient (5%)
Transfemoral access	19 patients (100%)
Valve size (mm)	
23	4 patients (21%)
26	12 patients (63%)
29	3 patients (16%)
No of post‐dilatations	
0	15 patients (79%)
1	4 patients (21%)

### 3.2 Borderline Indications Leading to PMI

**Figure 1:**
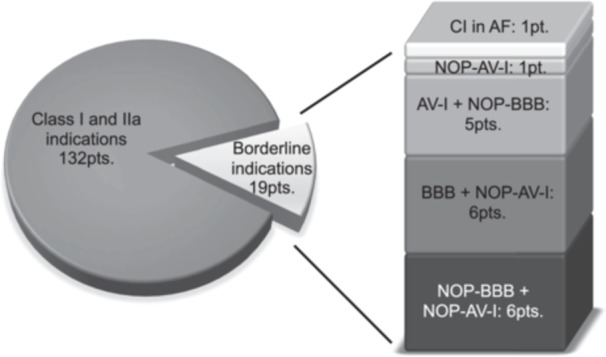
Borderline pacemaker indications leading to pacemaker implantation. AF, atrial fibrillation; AV‐I, first‐degree AV‐block; BBB, bundle‐branch‐block; CI, chronotropic incompetence; NOP, new‐onset and persisting.

Of 19 patients who received a pacemaker for not guideline‐supported indications, one patient received the PM for a new‐onset and persisting AV‐I (NOP‐AV‐I) with additive need for β‐blockers, 6 patients received the PM for NOP‐AV‐I with previous BBB, 5 patients had NOP‐BBB and previous AV‐I, 6 patients had NOP‐BBB and NOP‐AV‐I, 1 patient received a PM due to new‐onset atrial fibrillation with chronotropic incompetence. One patient had an electrophysiology study to evaluate the HV interval after he developed an AV‐I on top of a previous LBBB. He received a PM in the setting of a prolonged HV interval of 70 ms. The indications leading to PMI are presented in Figure 1. The CA mainly developed several days after TAVI with their peak leading to the decision for a PM averaging at 5 ± 2.5 days. PM‐implantation was performed at 7 ± 2.5 days after TAVI.

### 3.3 Device Choice and PM Programming

Of the 19 patients who underwent PM implantation 16 patients received a two‐chamber pacemaker and 3 patients got a single‐chamber pacemaker. In most of the cases, the pacemakers were programmed to achieve a minimum of ventricular pacing; therefore, DDD‐ADI, PR‐interval above 220 ms, and long lower rate intervals were chosen.

### 3.4 Pacemaker Dependency

**Figure 2:**
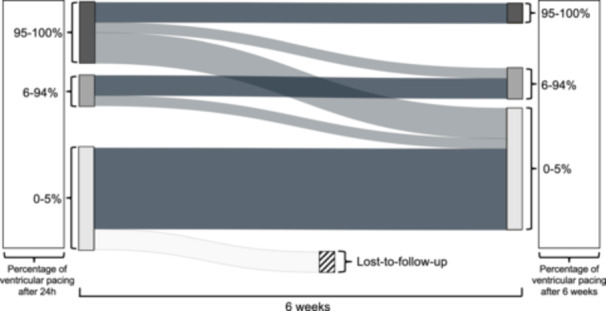
Sankey‐diagram of the development of ventricular pacing amounts between the first control after 24 h and 6 weeks. To improve the illustration, the patients were stratified into groups of ventricular pacing amounts of 0%−5%, 6%−94%, and 95%−100%.

Twenty‐four hours after implantation, the average pacing amount was 37.4 ± 46.2%. Whereas 6 patients had a high pacing amount (95%−100%), 2 patients had a medium pacing amount (6%−94%), and 11 patients had a low to no pacing amount (0%−5%). All patients had an intrinsic ventricular rhythm > 30/min. After 6 weeks, 2 patients had a high pacing amount, 3 patients had a medium pacing amount, and 12 patients had a low to no pacing amount. Two patients were lost‐to‐follow‐up, as they did not return to our center for further follow‐up visits. The 2 patients with high pacing amounts had preinterventional RBB with postinterventional AV‐I and preinterventional AV‐I with postinterventional LBB and progressing AV‐I to 360 ms. The temporal changes in the pacing amounts are presented in the Sankey diagram in Figure 2 and Table 2. In short, 8 patients showed decreasing pacing amounts while no patient had an increasing pacing amount between 24 h after implantation and the 6‐week visit.

**Table 2:** Patient characteristics, ECG features, PM programs, and follow‐up.

Patient characteristics	Preinterventional CAs	Postinterventional CAs	Onset or peak of CAs (*d*)	PM‐mode ventricular rate PR‐interval (sensed‐paced)	Pacing amount after 24 h (%)	Pacing amount after 6 weeks (%)	Intrinsic rhythm > 30 bpm at 6 weeks
84‐year‐old female	None	AV‐I to 320 ms and recurrent SVT‐episodes with need for β‐blockers	7	DDD‐ADI VR: 50 bpm PR: 190–110 ms	1	1	✓
75‐year‐old female	AF and LAH	Chronotropic incompetence under AF to maximum 70 bpm	2	VVIR VR: 50 bpm	100	18	✓
83‐year‐old female	RBBB	Progredient AV‐I to 300 ms	8	DDD‐ADI VR: 50 bpm PR: 200–170 ms	5	Lost‐to‐follow‐up	
85‐year‐old male	None	LBB, AV‐I to 230 ms	2	DDD VR: 50 bpm PR: 275–250 ms	100	0.1	✓
74‐year‐old male	RBBB	Progredient AV‐I to 280 ms	7	DDD VR: 50 bpm PR: 180–140 ms	100	100	✓
86‐year‐old female	AV‐I to 220 ms	LBBB, progredient AV‐I to 360 ms	4	DDD VR: 60 bpm PR: 210–170 ms	100	100	✓
81‐year‐old female	None	LBBB progredient AV‐I to 250 ms	4	DDD‐ADI VR: 50 bpm PR: 180–140 ms	< 1	< 1	✓
83‐year‐old female	AV‐I to 210 ms	LBBB progredient AV‐I to 260 ms	10	DDD VR: 50 bpm PR: 330–310 ms	< 1	< 1	✓
78‐year‐old male	AV‐I to 210 ms	LBBB progredient AV‐I to 300 ms	6	DDD‐ADI VR: 60 bpm PR: 180–140 ms	< 1	< 1	✓
85‐year‐old female	LBBB	AV‐I to 430 ms	4	DDD VR: 50 bpm PR: 250–230 ms	40	< 1	✓
83‐year‐old female	AV‐I to 230 ms	LBBB progredient AV‐I to 290 ms	4	DDD VR: 50 bpm PR: 150–140 ms	21.3	14	✓
84‐year‐old male	None	LBBB progredient AV‐I to 300 ms	10	DDD VR: 50 bpm PR: 300–250 ms	5	4	✓
86‐year‐old female	None	LBBB progredient AV‐I to 250 ms	7	DDD VR: 50 bpm PR: 200–150 ms	5	Lost‐to‐follow up	
93‐year‐old female	None	RBBB, LAH, and AV‐I	4	DDD‐ADI VR: 50 bpm PR: 180–140 ms	99	1	✓
76‐year‐old female	None	LBBB progredient AV‐I to 250 ms	4	DDD VR: 50 bpm PR: 200–150 ms	40	31	✓
80‐year‐old male	LBBB	AV‐I to 250 ms, HV‐interval 60−70 ms	2	DDD VR: 50 bpm PR: 180–140 ms	100	1.7	✓
82‐year‐old female	LBBB	New onset AV‐I to 310 ms	5	VVI VR: 40 bpm	< 1	< 1	✓
87‐year‐old male	AV‐I to 210 ms and RBBB	Progredient AV‐I to 280 ms	5	DDD‐ADI VR: 50 bpm PR: 180–140 ms	5	5	✓
84‐year‐old female	AV‐I to 240 ms	LBBB	2	DDD VR: 50 bpm PR: 200–150 ms	1	1	✓

Abbreviations: AF, atrial fibrillation; AV‐I, first degree AV‐block; BBB, bundle‐branch‐block; CA, conduction abnormality; CI, chronotropic incompetence; LAH, left‐anterior hemiblock; NOP, new‐onset and persisting; PM, pacemaker; PR, PR‐interval; VR, ventricular rate.

Of the 12 patients with low pacing amounts, 10 patients had PM modes to reduce ventricular pacing, long PR‐delays, or low base rates. The two patients with high pacemaker dependency had no PM modes to reduce ventricular pacing or PR‐delay ≤ 210 ms activated.

### 3.5 Evolution of Ejection Fraction 1 Year Post‐TAVI

Twelve of 19 patients had an echocardiographic follow‐up in our center after 1 year. Of these, no patient developed a significant decrease in ventricular function regardless of the amount of ventricular pacing.

## 4 Discussion

The still higher rate of PM implantations after TAVI compared to surgical valve replacement remains a concern. While high‐grade CAs (especially third‐degree AV‐block) with consecutive clear pacemaker indications are dominating, borderline indications remain a challenging aspect of postinterventional care. Especially progressively prolonging AV‐conduction times are suggestive for the development of high‐grade AV‐blocks and the consecutive risk of syncope or cardiac arrest. Important to note in this context is that the new 2021 guideline also provides a class IIa indication for PMI in the case of preexisting RBBB and newly occurring conduction disturbances [10].

### 4.1 Safety of PMI

PMI was safe and without severe intra‐ or postoperative complications. No patient had device‐related complications within the first 6 weeks after implantation, no pneumothorax occurred, and no device‐related endocarditis was reported to our center. Echocardiographic controls at 1 year after TAVI did not show a negative impact of PM‐implantation or high left ventricular pacing percentages on left‐ventricular function. However, a recent meta‐analysis reported a higher all‐cause mortality and rehospitalization rate at 1 year for patients requiring PMI after TAVI [13] and even more concerns remain for long‐term outcomes. Thus, unnecessary PMI should definitely be avoided.

### 4.2 Ventricular Pacing Percentages and Pacemaker Dependency

Evaluation of PM dependence is difficult as pacing amounts do not necessarily represent the degree of PM dependence. On the one hand, high ventricular pacing amounts can result from PR‐interval programmed shorter than the intrinsic AV‐delay despite intact AV‐conduction. On the other hand, even infrequent ventricular pacing can result from short episodes of complete heart block.

When focusing on the patients with high pacing amounts at 6 weeks after PMI, both patients had PR‐interval programmed to a shorter duration than their intrinsic AV‐delay even before TAVI and algorithms to avoid ventricular pacing were not activated. Keeping this in mind, these patients cannot generally be declared as PM‐dependent.

Among the patients who showed reduced pacing amounts, 3 patients had programs to reduce ventricular pacing (DDD‐ADI, long PR‐interval) and 2 patients had decreasing pacing amounts with strict pacing PM programs which can be seen as a clear sign of a recovering conduction.

Furthermore, as 10 of 12 patients with decreasing pacing amounts had pacing‐reducing PM modes activated, these programs seem to be highly effective in reducing ventricular pacing amounts in borderline collectives.

Previous studies investigating PMI in borderline indications after TAVI showed heterogeneous results. Knecht et al. [14], who used an electrophysiology study‐guided approach to manage PMI decisions in LBBB after TAVI reported high rates of PM dependence of about 50% in patients with HV‐interval > 55 ms while defining PM dependence as a minimum of 1% ventricular pacing, which is a rather low cut‐off. Furthermore, the same group reported very low rates of PM‐dependency in a patient group with LBBB or LBBB plus AV‐I with a stricter definition of PM‐dependence [11]. In addition, different kinds of prosthesis were used in these studies, of which some show drastically higher rates of postinterventional CAs than the Edwards 3 valve [15].

All in all, we consider pacemaker dependence as probably low as all patients had intrinsic rhythm > 30 bpm after 6 weeks and the majority of patients showed significantly declining or constantly low pacing amounts. Furthermore, no patient showed an increasing pacing amount which could be considered as a sign for progressive CA.

### 4.3 Pacemaker Benefits Beyond PM‐Dependency

Although individual patients do not show PM‐dependency, very long PR‐intervals, tend to be unphysiological and result in negative effects on ventricular function [16]. In the setting of a compromised diastolic function, which is often found in TAVI collectives [17], the negative influence of a prolonged PR‐interval with atrial and ventricular asynchrony particularly come into play [18] and, therefore, the possible positive effects of a PM‐implantation have to be taken into consideration in individual patients.

## 5 Limitations

This study focused on patients with borderline CA leading to PMI after TAVI. The key limitations of this study are due to retrospective data analysis and the relatively small patient population. As mentioned above, it is very difficult to evaluate the pacemaker dependency from PM memories retrospectively, but this study was able to show that progressively prolonging first‐degree AV‐blocks or new‐onset BBB do not tend to become persisting high‐grade AV‐conduction delays. Furthermore, previous studies [14, 19, 20] focused on the value of electrophysiology studies after TAVI to evaluate the potential prognostic need of PMI but these studies are all together limited to small sample sizes, differences in EPS‐timing, and different cut‐off‐values so that no general recommendation for EPS to guide PMI in patients with borderline indications for PMI can be given.

## 6 Conclusion

Borderline CAs are not uncommon after ballon‐expandable TAVI and their management remains controversial. In our cohort of 1083 TAVI patients, 19 patients underwent PMI for borderline CAs (11.5% of all PMI). Of these, only 2 patients had high pacing amounts after 6 weeks. This suggests that these CAs are often recovering over time and that the risk of complete persisting heart block in this smaller collective seems to be low. Furthermore, algorithms to reduce ventricular pacing are highly effective to avoid ventricular pacing whenever reasonable.

## Conflicts of Interest

Helmut Baumgartner: Congress travel support: Edwards Lifesciences, Abbott, Medtronic; Speaker fees: Edwards Lifesciences. Fernando De Torres‐Alba: Congress travel support: Edwards Lifesciences. Gerrit Kaleschke: Congress travel support: Edwards Lifesciences. Gerrit Frommeyer: Congress travel support: Abbott, Medtronic; Fellowship participant: Boston Scientific. Christian Ellermann: Congress travel support: Abbott, Medtronic; Fellowship participant: Boston Scientific. Florian Reinke: Speaker and advisor fees: Medtronic, Boston Scientific; Speaker fees: Biotronik. Lars Eckardt: Congress travel support: Abbott, Medtronic. Julian Wolfes: Fellowship participant: Boston Scientific. The other authors declare no conflicts of interest.

## Data Availability

Data are available on reasonable request from the authors.
